# Meta-omics data and collection objects (MOD-CO): a conceptual schema and data model for processing sample data in meta-omics research

**DOI:** 10.1093/database/baz002

**Published:** 2019-01-31

**Authors:** Gerhard Rambold, Pelin Yilmaz, Janno Harjes, Sabrina Klaster, Veronica Sanz, Anton Link, Frank Oliver Glöckner, Dagmar Triebel

**Affiliations:** 1 University of Bayreuth, Universitätsstraße 30, Bayreuth, Germany; 2 Max Planck Institute for Marine Microbiology, Celsiusstraße 1, Bremen, Germany; 3 Jacobs University, Campus Ring 1, Bremen, Germany; 4 SNSB IT Center, Menzinger Straße 67, München, Germany

## Abstract

With the advent of advanced molecular meta-omics techniques and methods, a new era commenced for analysing and characterizing historic collection specimens, as well as recently collected environmental samples. Nucleic acid and protein sequencing-based analyses are increasingly applied to determine the origin, identity and traits of environmental (biological) objects and organisms. In this context, the need for new data structures is evident and former approaches for data processing need to be expanded according to the new meta-omics techniques and operational standards. Existing schemas and community standards in the biodiversity and molecular domain concentrate on terms important for data exchange and publication. Detailed operational aspects of origin and laboratory as well as object and data management issues are frequently neglected. Meta-omics Data and Collection Objects (MOD-CO) has therefore been set up as a new schema for meta-omics research, with a hierarchical organization of the concepts describing collection samples, as well as products and data objects being generated during operational workflows. It is focussed on object trait descriptions as well as on operational aspects and thereby may serve as a backbone for R&D laboratory information management systems with functions of an electronic laboratory notebook. The schema in its current version 1.0 includes 653 concepts and 1810 predefined concept values, being equivalent to descriptors and descriptor states, respectively. It is published in several representations, like a Semantic Media Wiki publication with 2463 interlinked Wiki pages for concepts and concept values, being grouped in 37 concept collections and subcollections. The SQL database application *DiversityDescriptions*, a generic tool for maintaining descriptive data and schemas, has been applied for setting up and testing MOD-CO and for concept mapping on elements of corresponding schemas.

## Introduction

In ‘omics’ research approaches, massive parallel sequencing technologies [‘High-Throughput Sequencing’ (HTS)] provide insights into entire genomes of individual organisms, populations of species and the phylogenetic or functional structure of whole communities of microbial organisms. In addition, advanced mass spectrometry and nuclear magnetic resonance (NMR) spectroscopy technologies can further be applied to assess metabolite profiles of environmental samples (1, 2, 3). Omics disciplines comprise genomics, transcriptomics, proteomics as well as metabolomics, referring to the genome, transcriptome, proteome and metabolome, respectively, of a species, population of species or community of species.

For the cultivation-independent analyses of environmental samples or microbiomes, the disciplines of ‘metagenomics’, ‘metatranscriptomics’, ‘metaproteomics’ and ‘metametabolomics’ have been introduced (4). They are frequently referred to as ‘community genomics’ or summarized as meta-omics (5). These new processing and analysis techniques opened an unprecedented approach to study complex ecosystems, also involving biogeochemical processes, host organism metabolism and pathogenicity (6–9).

The α- and β-diversity of microbial communities can be assessed by analysing rRNA gene sequences (16S in bacteria, 18S in eukaryotes), and the internal transcribed spacer, typically in fungi (10, 11, 12), either by cultivation-independent amplicon, shotgun metagenome or metatranscriptome sequencing. Such analyses are based on total DNA or RNA extracts of microbiomes with subsequent short-read sequencing of the entire mixture. The resulting millions of short random DNA/cDNA fragments may be (partially) assembled or used individually as markers for specific organisms and metabolic functions. Compared to rRNA gene amplicon sequencing, shotgun meta-omics typically provides insight into the functionality of microbes and their biological processes, including horizontal gene transfer, sequence variants and evolutionary variability and genome plasticity (13).

The common application of the aforementioned techniques and the accompanying accumulation of meta-omics data provide unprecedented insights into the phylogenetic and functional diversity of microbial communities (14). While comparatively studying microbiomes in time and space, meta-omics data need to be shared within projects and among researchers early in the data lifecycle (15). In order to be able to derive conclusions from shared and combined meta-omics datasets, it is essential that research data follow the FAIR data principle, which means Findable, Accessible, Interoperable and Reusable (16, 17), and are made comparable and transferable across platforms and different big data study approaches (18).

Comparing data and datasets from different experimental approaches as well as different data processing platforms and workstations is challenging, as the degree to which different data are provided with background information typically varies between the projects and workgroups. It is often a lack of essential structural details combined with a lack of early data curation of analysis results that hinders the correct interpretation of the complex information provided in primary datasets (19). Large and complex microbial–ecological studies often rely on the complementation of different kinds of meta-omics data, where taxonomic and functional data are combined in order to compensate for incomplete databases (20). In this context, it becomes apparent that meta-omics datasets from different sampling strategies and research approaches need—at a minimum—standardized metadata and taxonomic identification data (21). To be reusable in the sense of FAIR, however, they need to be structured according to rich and well-documented data processing and exchange schemas.

With the above-mentioned advance of methods and analysis tools in meta-omics studies, the research process is usually divided into various operational steps along sophisticated work- and dataflows, based on teamwork and job sharing. This highlights the need for the establishment and documentation of these steps and of corresponding data pipelines starting with the acquisition of samples in the field, and for documentation of identifiers at the start of data generation (22). The need for new approaches for the design of data models and applications for data processing is evident; however, the underlying theory of data processing also needs to be expanded by developing conceptual schemas and operational standards.

## Conceptual schemas and their mapping

‘Conceptual schemas’, as defined in information science, are high-level descriptions of information and typically include concepts (23) with pre-defined values, and the most relevant relationships between them. Such schemas are preconditional for building data models for the implementation of database systems, as well as a basis for database record transfer and merging tools. They are also required for data interpretation and recovery from legacy software and for database-independent long-term data preservation and archiving. Conceptual schemas provide terminologies and ontologies that have either been formally accepted in a standardization process or just represent a *de facto* standard by common application. They represent a map of concepts and are often extended to build a (entity-relation) ‘data model’. The latter explicitly determines the structure and relationships of data used for implementation issues.

Conceptual schemas and data models, respectively, are subject of research in biological informatics. They are set up and applied by different life science communities for various primary goals: (i) for structuring and modelling of biological information on environmental observation, sampling events and storage of physical collection objects; (ii) for representing operational lines with descriptions of objects and protocols along work- and dataflows; (iii) for linking, mapping and transferring database contents; and (iv) for establishing or addressing ontologies in the semantic web context.

New developments in meta-omics research and analysis already induced the establishment of minimum reporting guidelines and lists of concepts and parameters, of database information models and related data exchange schemas (see chapter ‘Discussion’ below). Since activities of natural science collections and biobanks increasingly overlap with meta-omics analysis, some of the existing data exchange schemas have been extended accordingly, for instance, ABCDGGBN-Enviro (http://data.ggbn.org/schemas/ggbn/Enviro/ABCDGGBN_Enviro.html) (24) and Minimum Information about any Sequence (MIxS) (25). Most existing conceptual schemas in biology, however, focus on data exchange and data publication. Correspondingly, they address the interlinking of repositories in the ‘downstream’ section of the data life cycle. They are less suitable for covering operational aspects like field and laboratory processing as part of the workflow towards data production, i.e. the ‘upstream’ section of the data life cycle (26).

At this point, it should be mentioned that conceptual schemas are comprehensive and structured concept collections for facilitating the organization of data elements and relations between them for a broader research community. They are a subject of standardization committees and ideally commonly accepted by a large community. They are created for the purpose of facilitating technical communication and interoperability issues. This kind of schema should not be mixed up with ‘organized lists of parameters and elements’ in use by several project internet platforms. One example is Qiita, a pipeline to organize research data of the microbiome community with the aim to aggregate these for meta-analyses. Parameters as used in such platforms are not intended to build a comprehensive schema for being approved as standard for general data processing and data exchange in meta-omics research (27). They might, however, influence schema development by providing vocabulary elements.

Standard conceptual schemas in biology hitherto typically rely on ‘text and numerical data types’. Text data types allow for a high flexibility regarding their content. A mapping between two schemas with similar concepts but without pre-defined conceptual values is relatively easy, but such flexibility might result in heterogeneity of the stored data regarding the values assigned to a concept. As a consequence, such data requires additional efforts during preceding and subsequent data maintenance, especially if further data mapping and transformation is envisaged. The efforts to improve data quality may, for instance, concern the secondary linking of values with thesauri, taxonomies and vocabularies. Thus, the result might be sufficient to allow for sensible data mining, statistics, visualization and the use of data sets for Linked Open Data (LOD).

‘Schema mapping’ and data transformation issues might address several levels of abstraction, those of the conceptual schemas, their concepts (‘elements’, ‘descriptors’) and concept values, and those of the data models with their relations between entities. To optimize the results of data transfer, it is advantageous that the source schema includes a number of ‘concepts with predefined values’, which are less challenging during data merging operations. The categorical data type is usually applied for describing qualitative properties and allows for a ‘controlled terminology of concept values’ (28). However, the use of such predefined values (‘descriptor states’) in the target schema implies constraints with regard to the need to exactly match the source schema element values (categories) during schema mapping. In such cases, source schema elements and values, which are not represented in the target schema and cannot be mapped, may be assigned either to a predefined default supplementary property (‘other value’) or, like to any element of text data type, onto the respective target schema element as a whole, with the original wording of the source data element value. Additional options of concept assignment concerning type and degree of matching include the use of Simple Knowledge Organization System (SKOS) specifications.

The new conceptual schema described here is addressing challenges in the management and transformation of complex processual data as created in meta-omics research. It has a sufficiently generalized logical structure with consistent concepts and concept values, pertaining to various concept collections, and addresses constraints of schema mapping with existing biodiversity data standards.

## The MOD-CO schema

MOD-CO stands for ‘Meta-omics Data and Collection Objects’. The schema has been designed to close existing gaps by addressing both the upstream and downstream sections of the data life cycle. It addresses operational aspects including storage, as well as traits of objects in meta-omics research, and is created as a structural and terminological backbone for data processing in research groups, biobanks and life science collections. It may support the representation of individual objects and operations as well as of entire work- and dataflows. MOD-CO includes a high number of concepts with predefined values (categories).

**Figure 1:**
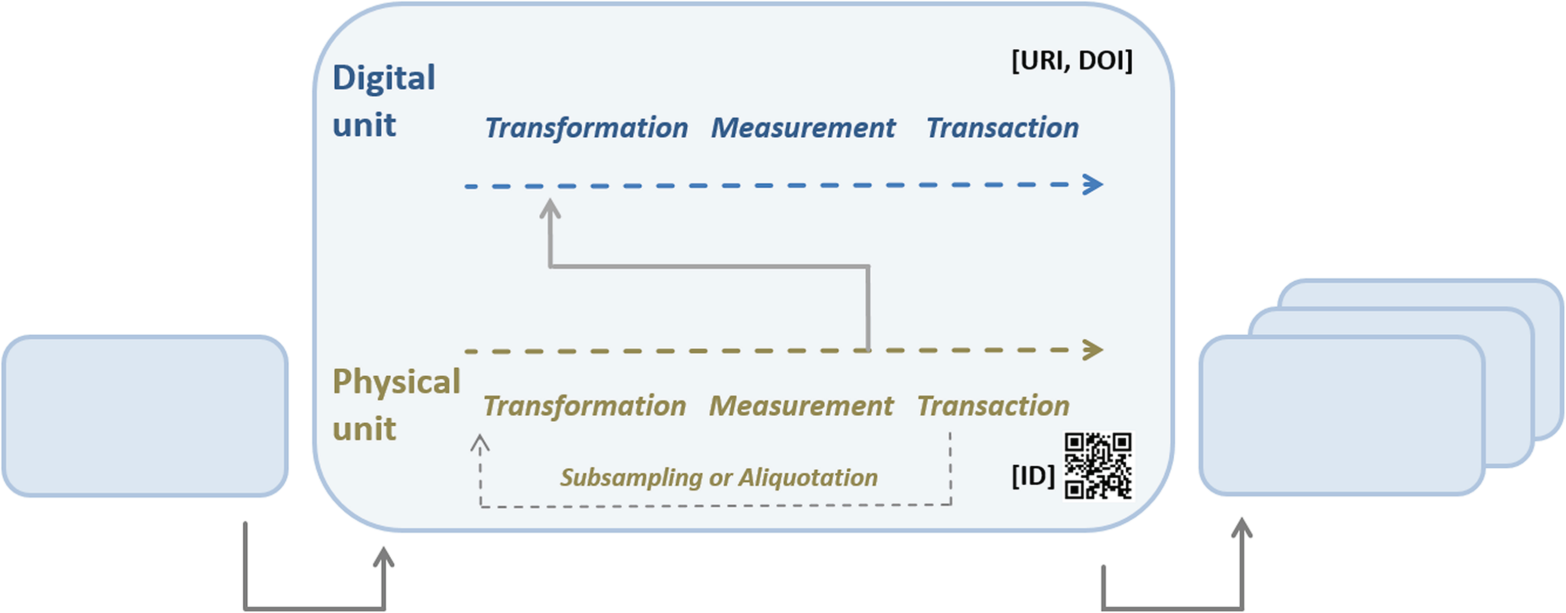
Workflow segments concatenated to a workflow. A workflow segment comprises the elementary operations transformation, measurement and transaction being applied once or twice (due to preceding subsampling or aliquotation) to a physical object in focus, and the generation of data (measurement) and its subsequent transformation, measurement and transaction within the segment.

MOD-CO focuses on the entire operational workflow from the researchers’ or laboratory technicians’ points of view. It operates with interlinkable identifiers referring to the unit (i.e. physical or digital object) itself, to the preceding unit (or an aliquot, i.e. subsample thereof), and to the origin or initial unit, which may be the original sample gathered in the field. This allows for a concatenation of data records representing the individual workflow segments. Each workflow segment usually starts with subsampling or aliquotation (i.e. transformation) of a physical unit, or product or sample of the preceding segment, followed (or not) by measurement. Products, having subsequently been transformed and measured according to a laboratory protocol, are usually translocated and deposited in a container (transaction). Measurement data, usually generated for quality control, are stored (transformed), analysed and interpreted (measured) and archived (transacted) as well (Figure 1).

In its version 1.0, first released on 28 March 2018, MOD-CO comprises 653 concepts. When compared with other schemas, it (i) includes a considerable number of elements, relevant for meta-omics data work- and dataflows; (ii) allows for describing such work- and dataflows by concatenated records of workflow segments (from sampling in the field to publication); and (iii) defines the endpoints for feeding URI identifiers for addressing web resources, i.e. vocabularies, namespaces or multimedia objects, as well as concept URIs of other schemas and data models.

### MOD-CO concepts, data types and relations

The 653 schema concepts are classified as

- text (‘<string>’) data type (398 concepts),
- categorical (‘<category>’) type (238, with 1810 predefined concept values or states in total),
- quantitative (‘<value>’) data type (7 concepts) and
- sequence (‘<string>’) type (10 concepts).

Quantitative data types may include measurement as well as time–space descriptors. In addition to the selection of predefined values or states, categorical concepts or descriptors may also require additional free text specification (‘<category>’ or ‘<category> <string>’, respectively).

**Figure 2:**
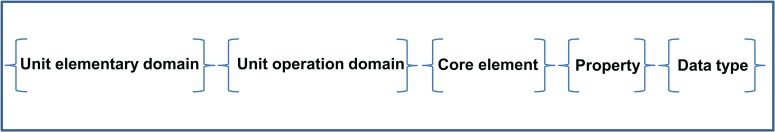
Naming of MOD-CO concepts according a multipartite approach.

‘The naming of MOD-CO concepts’ follows a multipartite structure. This convention includes (i) the reference to unit ‘elementary’ domain, (ii) the reference to unit ‘operation’ domain, (iii) a ‘core element’, (iv) a specific ‘property’ (29, 30) and (v), as the final segment, the ‘data type’ (e.g. for text: ‘<string>’). Core elements may be agents, i.e. persons and organizations, devices, organisms or parts thereof. Properties may concern ‘presence’, ‘name’, ‘ID’, ‘notes’, ‘URI’, etc. (Figure 2). The preceding unit elementary domain is ‘physical’ or ‘digital’ and the unit operational domain is transformation, measurement or transaction (Figure 1).

Example 1: The concept ‘unit digital measurement device URI <string>’ is therefore composed of unit elementary domain: unit digital—unit operation domain: measurement—core element: device—property: URI—data type: <string>.

Example 2: The concept ‘unit physical transformation community genomic analysis sequencing library index primer name <string>’ is composed of unit elementary domain: unit physical—unit operation domain: transformation—core element: community genomic analysis sequencing library index primer—property: name—data type: <string>.

Example 3: The concept ‘unit physical (host) organism muscle tissue <category>’ is composed of unit elementary domain: unit physical—unit operation domain: n/a—core element: (host) organism muscle tissue—property: n/a—(predefined) values (descriptor states): [cardiac, skeletal, smooth muscle, other value]—data type: <category>.

The operation domain ‘Transformation’ refers to any human-planned, more or less invasive treatment of an object that results in the case of a physical unit in fragmentation or dissection, shape or consistency change, chemical reaction or in the case of a digital unit in a new representation of data or data format.

‘Measurement’ refers to any more or less non-invasive allocation and comparative examination and analysis of a given object or unit by some objective method, concerning quantity, size, consistency or magnitude, and results in ordinal or quantitative values. If appearing to have been invasive, a transformation step may just have preceded measuring and may be formalized accordingly. Measurement also applies to analysis of data and the creation of information and interpretation from existing data, such as the creation of image parameter values by image pixel analysis.

‘Transaction’ refers to any shift, movement or transport of a physical object, such as from the field into the laboratory, the deposition of vouchers in storage containers or the transfer of samples to other places for processing, including an external institution. Correspondingly, this domain refers to the transfer of data from one medium to another internal or external storage or data storage unit for archiving, or to an information portal for the purpose of data publication.

The option of cross-linking single data records by various types of IDs allows for sequential (and reticular) ‘concatenation’ or ‘linking of objects’ (‘units’) according to the operational segments during a work- and dataflow (Figure 1). In particular, the schema addresses meta-omics operations on environmental samples. Objects in focus are either physical or digital ones. ‘Physical objects’ usually concern environmental samples, e.g. host organisms harbouring microbial communities, or parts thereof (e.g. DNA), amplicons thereof, e.g. PCR (polymerase chain reaction) products, and pools thereof, as well as derivatives like clone libraries. ‘Digital objects’ may concern measurement data on extracted DNA and amplicons, derived data on nucleic or amino acid sequences, secondary/tertiary macromolecular structures, as well as other derived data and information like sequence alignments, cladograms, statistics and visualizations thereof.

Although data record linking does not relate to the schema itself, this aspect is part of the MOD-CO data model and emphasized in an own chapter further below. The reason is to clarify the role of the preceding unit identifier. It also justifies certain redundancies of predefined concept values in cases when an individual data record refers to several or all segments of a workflow with various objects and operations to be described.

### MOD-CO concept collections

Schema concepts and values are assigned to ‘concept collections’ according to the LOD terminology (31).

The collection assignment or classification might be achieved in alternative ways and is not a mandatory part of a schema. However, it can be a guideline for implementation.

In schema version 1.0, there are 37 concept (sub-)collections (= trees and subtrees) that means 13 concept trees with 8 of these subclassified into 24 concept subtrees in total (see, e.g. images under https://www.mod-co.net/wiki/Schema_Representations). The classification of concepts in 13 concept collections is described here shortly. The name of the 24 subcollections is indicated in square brackets.

MOD-CO concept collection: ‘Schema hierarchy’ [level 0—general: 61 concepts assigned—level 1—operation general: 277—level 2—object general: 49—level 3—operation specific: 215—level 4—object specific: 51]: In MOD-CO, concepts are hierarchically organized into five schema hierarchy levels (levels 0–4). Each concept is assigned to one level only. While the lower levels particularly concern elementary operational aspects or object properties, the higher ones characterize options at a higher specificity. Schema level 0 provides ‘general concepts’, e.g. the internal unit identifier, which represents the major key identifier. Other types of identifiers concern the relations to units within a workflow (e.g. ‘unit identifier preceding’ or ‘unit identifier origin’), as well external resources. Functional concepts at level 0 concern data record maintenance aspects, represented by ‘unit record creation date/time’ and ‘… agent’, etc. The type of unit being addressed (‘unit domain elementary’), i.e. a physical unit or a digital unit, is also specified at schema level 0. Finally, the ‘unit operation domain’ descriptor with the states ‘transformation’, ‘measurement’ and ‘transaction’ refers to the ‘elementary processes’ during a workflow. Schema level 1 contains a high number of ‘general operational concepts’ relevant in the context of transformation, measurement and transaction of physical objects or data. In contrast, Schema level 2 provides ‘general object-related concepts’ relevant in the context of physical object or data traits descriptions. Schema level 3 concepts allow for ‘specifying operational aspects’, while Schema level 4 concepts allow for specifying object and data or document properties (‘specific object-related concepts’). Redundancies of predefined values/states occur in some schema level 3 concepts, e.g. the states of ‘unit physical transformation protocol modification’ with the property identifier, parameters and specification, as well as the modification identifier, parameters and specification. Such redundancies of property terms or descriptor state terms may allow the joining of data of several workflow segments into one single data record, if required.

‘Identifiers’ [DOI: 2—ID: 82—Name: 158—URI: 66]: this collection refers to concepts representing an identifier, specific term or name for referring to a given sample, sample trait, data or document (physical and digital units). In addition, foreign identifiers (DOIs, IDs or URIs) may link to external resources. Such external IDs may refer to unit properties, applied methodologies, as well as contextual information, which are required for understanding the status of a given physical or digital unit. Concepts specifying general IDs concentrate at schema hierarchy levels 0, 1 and 3. Those specifying URIs, referring to locations, names and documents, are located at all schema hierarchy levels (0 to 4).

‘Space–time details and specifications’ [99 concepts assigned; no subcollections]: geographic coordinates, altitude, depth and date–time are the most relevant data for describing the origin of a sample and the location of sample processing. The concepts of the space–time-related aspects are therefore required in various contexts concerning sampling and sample processing as well as acquisition and analysis and result in the assembly of this concept in a concept collection of its own. They are mostly accompanied by concepts providing the formats for coordinates and date–time.

‘Unit property domains’ [Digital: 235—Physical: 380]: this collection includes concepts for ‘digital’ and ‘physical’ units (for definition see chapter above). A database structured according to MOD-CO will therefore allow for addressing the data, e.g. of physical objects like environmental samples, as well as the data of contextual digital units such as document files with analysis data by measurements, or published documents.

‘Unit operation domains’ [Transformation: 241—Measurement: 174—Transaction: 112]: this collection refers to the three elementary operational categories being ‘transformation’, ‘measurement’ and ‘transaction’, as defined in the chapter above. Such operations that a physical or digital object may pass through are considered elementary.

‘Operational details’ [Method: 49—Parameter: 20—Protocol: 23]: working plans, methods, parameters and protocols are grouped here. They are necessary for providing information on the operational context during the work- and dataflow from the field to the laboratory, to the repository and to the publication platform. Parameters and protocols concern the tools and procedures to process a physical or digital object during field work or in a laboratory or storage context.

‘Operational specifications’ [60 concepts assigned; no subcollections]: measurement units, scales and calibrations are grouped here. These concepts deliver necessary information for interpreting analysis and contextual data correctly. As measurement values are usually stored as numerical type data, operational specifications like measurement units may be provided as separate concepts.

‘Operational tools’ [Hardware: 63—Software: 54—Enzyme, reagent: 16]: devices or tools like hardware and software, but also organic catalysts like enzymes, are of relevance for performing elementary operations (transformation, measurement, transaction) in the various segments along a work- and dataflow. They are assigned to this concept collection.

‘Environmental object types’ [(Host) organism: 26—(Environmental) substrate: 15]: living and non-living (field) samples like living or dead organisms, parts thereof, or various kinds of substrates like soil or human-made substances, being targeted in meta-omics research are grouped here.

‘Environmental object traits’ [27 concepts assigned; no subcollections]: for describing properly the origin of an environmental sample, refined classifications of sample traits are required. The most relevant traits have been grouped as a concept collection of their own.

‘Agent-related aspects’ [81 concepts assigned; no subcollections]: this concept collection comprises person- and organization-related descriptors. It includes, for instance, the collectors of samples and staff responsible for identifying samples or image documentation, and for databasing or processing samples in the laboratory or in a repository. Responsibility of persons and organizations as agents also concern interinstitutional transactions of samples for processing or deposition, as well as of data handling in an administrative context (permits) and for the purpose of publication. A number of concepts at schema hierarchy level 3 provide the option to specify a ‘robot status’ of an agent.

‘Legal issues-related aspects’ [Agreement, compliance, permit: 21—Project: 5]: this collection has two subcollections with information necessary for handling samples and documents subject to legal aspects. They include agreements, permits and compliances on sampling and export as well as the assignment of environmental samples to a research project or storage service.

‘Data record management aspects’ [22 concepts assigned; no subcollections]: this set of descriptors does not directly refer to the properties of an object or document, nor the transformation or transaction of a physical or digital object, but concerns the management of the data record (data entry in a database) itself. The concepts comprise, for instance, data on the responsible persons for data entry, as well as the date of start of data entry, history of revisions and completion.

### Concatenation of MOD-CO-based data records

All physical or digital units generated during work- and dataflow or during work- and dataflow segments may be described by the operations that have been applied for their creation from preceding units. For instance, while an initial physical unit or sample may have been created based on the process of partitioning by using a certain mechanical sampling device, pure DNA has been generated from a subsample or aliquot by applying extraction and purification methods referenceable by a protocol, and by using a laboratory kit. The same applies to the generation of DNA amplicons and the creation of DNA sequence data. Concatenation (linking) of data records (database entries) representing one or more operational steps is enabled by assigning a ‘unit identifier preceding’ (parent relation) to a given data record of a given physical or digital unit. MOD-CO also provides a child relation identifier ‘unit identifier subsequent’, which may be used for addressing a subsequent product, for instance created via the pooling of objects.

Thus, MOD-CO allows for characterization of the relevant operational segments by documenting process details, including protocol specifications and the devices applied, as well as the traits of the resulting object or product. In this way, it makes the creation of coherent information on a given work- and dataflow possible. This is a precondition to use MOD-CO as a database model for implementation of a laboratory information management system (LIMS) or an electronic laboratory notebook (ELN).

### Schema management and curation in *DiversityDescriptions*

Currently, MOD-CO is maintained in *DiversityDescriptions* (DWB-DD), based on a network installation of the Diversity Workbench (32). DWB-DD is based on a generic relational data model for triple store data. It is a suitable SQL database application for schema maintenance and updating, following release cycles. The data model of DWB-DD is generic and has been published (33). The DWB-DD rich client provides many options to manage the internal structure of a schema and its content. It includes various features for editing the schema concepts and values; for managing names and naming conventions; for modifying their arrangement; for assigning descriptors to various kinds of hierarchies (‘descriptor trees’); for linking them to resources with definitions and explanations; and for mapping them onto related external and internal conceptual schemas and standards. The parallel management of several schema versions is possible. Furthermore, DWB-DD also allows for the flexible export of various kinds of schema representations and XML documents.

### MOD-CO schema publication

**Figure 3:**
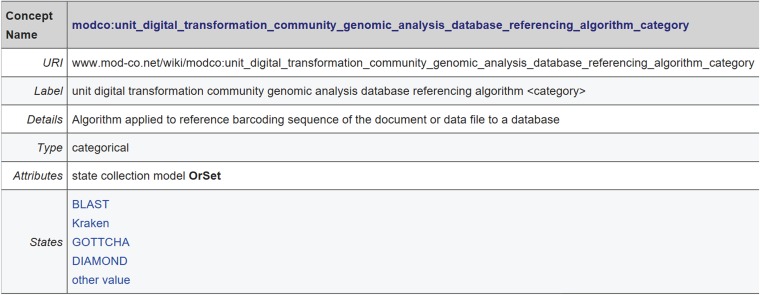
Excerpt of MOD-CO Schema Reference (versioned MOD-CO namespace scheme publication) with one concept showing concept name and concept details: the first elements of the multipartite name refer to the core element of the concept, the second to last (i.e. ‘referencing algorithm’) to its property and the last (i.e. ‘category’ equal to ‘categorical’) to the data type.

**Figure 4:**
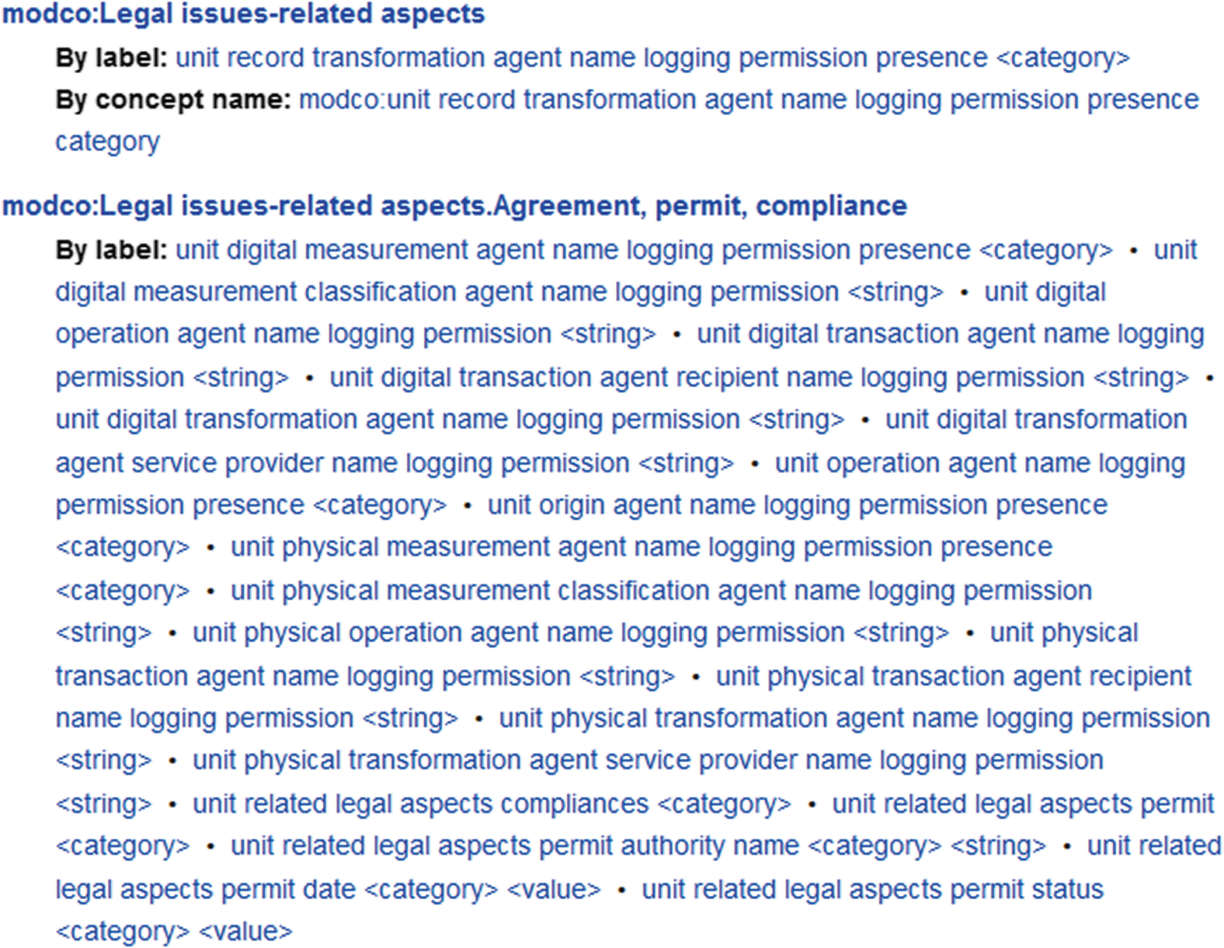
Excerpt of MOD-CO LOD-compliant namespace scheme publication with two concept collections and access to single Wiki pages, which provide stable URIs for each concept and concept collection.

The MOD-CO schema is currently published by export from DWB-DD, e.g. as XML for Semantic Media Wiki, SDD-structured XML (a community standard ratified by Taxonomic Databases Working Group (TDWG), today's Biodiversity Information Standards) and MOD-CO-structured XSD. The MOD-CO Wiki namespace ensures user-friendly accessibility to up-to-date versions of the schema at that representation level by linking stand-alone SQL database contents and Semantic Web representations with stable URIs (34, 35). In its current version, MOD-CO is available under https://www.mod-co.net/wiki/Schema_Representations:

(a) The versioned MOD-CO namespace scheme publication (static, snapshot) with stable URI under https://www.mod-co.net/wiki/MOD-CO_Schema_Reference (for an excerpt see Figure 3). Only minor editorial changes of content in the page body are possible. Content changes are documented under ‘View history’. This Wiki page also provides suggestions for citation.
(b) The LOD-compliant namespace schema under https://www.mod-co.net/wiki/MOD-CO with a stable URI string (http://www.mod-co.net/wiki/modco:) followed by the names of the 2463 concepts and values, 13 concept collections and 24 concept subcollections (in DWB-DD corresponding to 13 descriptor trees and 24 descriptor subtrees). The page design mainly follows the templates generated by the TDWG Terminology Wiki under https://terms.tdwg.org/wiki/, with few structural modifications (for an excerpt see Figure 4). Each MOD-CO concept is represented by a Wiki-page of its own, with stable URI and a link to Resource Description Framework (RDF; https://www.w3.org/RDF/) representation. The ‘issue date’ and each ‘modified date’ of the page content are accessible under ‘view history’. The contextual information in the page body for concepts includes definitions, cardinalities, data types, values and assignments to concept collections. In several cases, the relation of MOD-CO concepts to those of other schemas is given by the corresponding URIs. For definition and explanation purposes, the concepts are also linked to URIs from relevant internet resources such as Wikipedia, Obolibrary or Edam ontology. In future schema versions, the use of SKOS specifications will optimize the mapping on related schemas, thesauri, classifications and taxonomies.
(c) The SDD-structured XML representation for data exchange with other SDDcompliant databases (zip archive for download under https://www.mod-co.net/wiki/Schema_Representations). The file may directly be imported into a DWB-DD installation and be used as template for storing MOD-CO-compliant data in triple storage structure (for download of database and client, see https://diversityworkbench.net/Portal/DiversityDescriptions; for SDD documentation see above).
(d) A DWB-DD installation with MOD-CO version 1.0 is provided as part of the Diversity Workbench cloud network. This is done for training purposes.
(e) The structured normative XSD schema document under http://schema.mod-co.net/MOD-CO_1.0.xsd. This will facilitate validation of XML documents according to MOD-CO schema concerning well-formedness.

Feedback from the user community is welcome and will be temporarily added under the general feedback page and in the discussion page of each concept page in the MOD-CO Wiki.

### Schema usability for managing laboratory data

MOD-CO is ‘process-oriented’ with ‘hierarchically organized concepts’. The terminology of the generic concepts and predefined values is consistent and mostly based on a multipartite naming approach (see above). It is published as versioned representations with interlinked and persistent concepts, definitions and LOD-compliant stable URI identifiers. For implementing this schema as a logical backbone of a relational standard laboratory management system, the structure of the schema as described above is crucial but has to fulfil further criteria, which expand the logic of a classical conceptual schema and has to address the linking of data records (see section ‘Concatenation’ and Figure 1). A real-world use case for MOD-CO concerns the processing of fungal community samples and connected data in the context of the project GBOL 2. Details are provided on https://www.mod-co.net/wiki/GBOL_2_Fungi:_Microbiome_community_barcode_sequencing.

The data set comprises four segments of a fungal microbiome barcoding workflow from the sampling of leaves of rosaceous fruit trees in the field to total fungal nucleic acid extraction, to marker gene amplification and amplicon pooling and finally to HTS (MiSeq) sequence data generation. The use case data is provided as zip archive for download under a persistent SNSB ID: http://id.snsb.info/snsb/projects/1185. The version dated September 2018 contains one MOD-CO SDD-structured XML file (for research data), one EML-structured XML file (for research project metadata) and two CSV files, one for the EML data table and one for the GFBio compliant Dublin Core (DC) structured metadata.

As a consequence of the demands for maintaining the schema, the functional spectrum of the DWB module *DiversityDescriptions* has been extended. Recent versions of the software demonstrate its usability not only to manage and store conceptual and procedural hierarchical schemas in general, but also for use as a R&D LIMS with ELN functions.

## Discussion

### MOD-CO relationships to complementary schemas

In the fields of biodiversity research, molecular research and natural science collection domain, more than 30 community-agreed schemas and standards for data exchange and publication exist (see compilation on the GFBio Wiki—Concepts and Standards). Most of those are only used by a few major platforms as mentioned by Triebel *et al.* (36). Two major consortia exist, which are dedicated to the recommendation of standards in this field, i.e. the Genomic Standards Consortium (GSC) with the Biodiversity Genomics Working Group and the TDWG group on Biodiversity Information Standards. Four of these data exchange and data publication schemas or standards are Access to Biological Collections Data (ABCD), Darwin Core (DwC), GGBN and MIxS. They address a research community handling molecular data similar to that which MOD-CO does. Apart from the consortia GSC and TDWG, the Biobank Community for Biospecimens and their derivatives took over responsibility of developing the SPREC sample code with quality parameters as standard list (37) and the draft International Standard, ISO/DIS 20387 for biobanks (24).

The FAIRsharing portal provides links to 1133 standards for data publications, of which 160 are assigned to the subcategory ‘model/format standards’ in the domain ‘life science’. Only a few have machine-readable representations and are claimed as being standards. Very few have standard-compliant systems, such as databases, in place (38). This is the case for ABCD, DwC, MIxS and SDD. In the following sections, ABCD, DwC, MIxS and GGBN are briefly outlined and their relationships to MOD-CO are addressed.

‘ABCD with DNA Extension’: the ABCD 2.06 schema is a standard, ratified by TDWG in 2005 (39). It is devoted to the access to and exchange of data on specimens and observations (primary biodiversity data). The ABCD 2 schema is hierarchically structured and includes in its Linked Open Data compliant Wiki representation 1419 concepts with 15 concept collections in total. Version 2.06 is currently used by the Global Biodiversity Information Facility (GBIF) initiative, by European services like the Biological Collection Access Service for Europe networks, by Europeana and by several GFBio services. ABCD with thematic extensions is used to support portals like GeoCASe and BiNHum. With the ABCDDNA XML schema extension, it has been the starting point for setting up the schema for the GGBN portal. ABCD also comprises hierarchies and relations between terms. ABCD 2.06 is going to be collaboratively extended with ABCD 3.0. In comparison to MOD-CO, the data exchange standard ABCD 2 is less generic, less process-oriented and not meta-omics focussed. As its design dates back about 15 years, it has some logical and semantic constraints, which may be solved in version 3.0.

‘DwC’: DwC is a wide-spread standard, having been ratified by TDWG. It includes a glossary of terms (concepts), intended to facilitate the sharing of information about biological diversity by providing reference definitions, examples and commentaries. DwC is primarily focussed on taxa and specimens, samples, their occurrence and related information. DwC can be viewed as an extension of the DC for biodiversity information. It originally started with the biological specimen domain but now provides a stable standard reference for sharing information on biological diversity. DwC is a community-developed approach (40). A mapping of DwC to the ABCD standard exists. An experimental Linked Open Data representation exists for 270 elements or concepts, grouped in 15 concept collections. DwC is widely used as the data exchange standard in global biodiversity portals and initiatives as GBIF, iDigBio and EoL. It is checklist-oriented, less comprehensive than ABCD and MOD-CO, not process-oriented and not meta-omics focussed. As its design dates back about 15 years, it has some logical and semantic constraints.

‘MIxS’: the MIxS schema, developed and maintained by the GSC, consists of three separate minimum information standards (i.e. checklists): MIGS for genomes, MIMS for metagenomes and MIMARKS for marker genes. For creating a single-entry point to all minimum information checklists and to the environmental packages, an overarching framework was created and regarded as the MIxS standard with specifications (25). MIxS provides a method for introducing additional checklists and packages. The core MIxS team developed 15 environmental packages available as spreadsheets to annotate sample data. Adopters include the INSDC databases, which promote the use of MIxS elements or concepts during submission. The LOD representation includes 343 concepts with 5 concept collections. MIxS is relying on core vocabularies and thematic checklists for metadata publication. It is not developed to be comprehensive and process-oriented like MOD-CO. Many essential elements in this area are still missing.

‘Global Genome Biodiversity Network (GGBN)’: the GGBN data schema with its published standard specification (24) is a set of terms and controlled vocabularies designed to represent sample facts. It is based on the MIGS, DwC and ABCDDNA vocabularies (41) and not intended to be ratified as TDWG standard. It does not cover e.g. scientific names, geography or physiological facts. This allows combining the GGBN Data Schema with complementary standards such as DwC, ABCD or MIxS. Potentially, the schema can be used for non-human genomic samples as well as for human samples. It builds upon existing schemas and standards commonly used within the communities, extending them with the capability to exchange data on tissue, environmental and DNA samples as well as sequences. The GGBN Linked Open Data Wiki representation includes 156 concepts assigned to 11 concept collections, i.e. 11 vocabularies. These are compatible with MOD-CO concepts.

In comparison to the four data exchange schemas described above, the process-oriented MOD-CO conceptual schema is in several ways unique: it has a comparatively large number of concepts and predefined concept values and a hierarchical and consistently logical structure and is based on elementary object and operational types. The schema is less focussed on data exchange and data publication, but on the management of research data and process documentation. When compared to the schemas described above, MOD-CO is more comprehensive and structured in a way to serve as entity-relationship data model. Its provision of pre-defined values entails some constraints, which, however, makes it suitable for partially enforcing unambiguous mapping of information. Experimental mappings between MOD-CO on the one side and ABCD, DwC, GGBN and MIxS on the other are going on, and the results published under the respective concept entries (http://www.mod-co.net/wiki/Schema_Representations).

### Perspectives

MOD-CO has primarily been designed for the characterization of processes and objects during meta-omics analysis work- and dataflows, particularly the ‘upstream’ dataflow stage as described by Dallmeier-Tiessen *et al.* (26). Secondarily, it is suitable to foster the use of stable and persistent identifiers and other key components for facilitating high-quality data management and publication according to best practice reference models (19, 42, 43). MOD-CO concepts and values have been experimentally mapped onto other ontologies and schemas. This approach will be extended to cover all mandatory and non-mandatory concepts listed in a recent overview of the metagenomics standard environment (44). The mapping of modifiers for quantifications by use of the SKOS mapping property terms is going to be realized in an upcoming version of MOD-CO.

By continuation of mapping efforts between MOD-CO and the major biodiversity data exchange standards, the schema may eventually be applied as the core ontology of a transformation tool for data and metadata exchange, with a focus on meta-omics research. Feedback from the user community is welcome and will be added to a feedback page and to the discussion page of each concept page in the MOD-CO Wiki.

MOD-CO can be used as the logic backbone of R&D laboratory information and may also be applicable as core schema for a transformation tool between commercial LIMS and ELN software products like BioloMICS with other evolving community meta-omics-focussed schemas.

The database DWB-DD can be installed with the MOD-CO schema (or parts of it) as data model. DWB-DD has already a number of options for data transformation included and can act as a LIMS. The first real-world use case for MOD-CO compliant processing of data on microbiome community barcode sequencing in DWB-DD is already on the way to be published. This is achieved by using GFBio data pipelines with persistent ID as identifier (45). Common use of DWB-DD with implemented MOD-CO schema might strengthen it as a future ‘de facto standard’.

As communicated recently in an Editorial by Nature “… the microbiome community places great value on open data but, as a relatively young field, is struggling to establish standards …” (46). MOD-CO, as comprehensive conceptual schema for work- and dataflows from the upstream to the downstream stages in the meta-omics research cycle is a step forward in this direction. Its further development will rely on the expertise of a group of meta-omics and collection researchers. For a later version, formal standardization of the proposed schema may be envisaged.

## Notes

**Database URL:** http://www.mod-co.net/wiki/Schema_Representations

## Web links and abbreviations

(In alphabetical order; all URLs last accessed at July 27th, 2018)
ABCD Schema 2.06—: ratified TDWG Standard: http://www.bgbm.org/TDWG/CODATA/Schema
ABCD Schema 2—: LOD compliant Wiki representation: https://terms.tdwg.org/wiki/ABCD_2
ABCD 3.0—: Access to Biological Collection Data: https://abcd.biowikifarm.net/wiki/Main_Page
BiNHum—: Biodiversity Network of the Humboldtring: http://wiki.binhum.net
BioloMICS: https://www.bio-aware.com/
DC—: Dublin Core Metadata Initiative: DCMI Specifications: http://dublincore.org/specifications/
Diversity Workbench—: Training Environment: https://diversityworkbench.net/Portal/DWB_training_environment
Diversity Workbench—: Training Materials: https://diversityworkbench.net/Portal/Training_materials
DwC—: Darwin Core Introduction:http://rs.tdwg.org/dwc/
DwC—: LOD compliant Wiki representation: https://terms.tdwg.org/wiki/Darwin_Core
GBIF Resources—: Global Biodiversity Information Facility: technical resources: http://rs.gbif.org/
DwC Terms—: Darwin Core Terms: a quick reference guide: http://rs.tdwg.org/dwc/index.htm
Edam ontology: http://edamontology.org
ELN—: Electronic Laboratory Notebook: https://en.wikipedia.org/wiki/Electronic_lab_notebook
EML—: Ecological Metadata Language: http://www.dcc.ac.uk/resources/metadata-standards/eml-ecological-metadata-language
EoL—: Encyclopedia of Life: global access to knowledge about life on Earth: http://eol.org/
Europeana Collections: http://www.europeana.eu
FAIRsharing—: A curated, informative and educational resource on data and metadata standards, inter-related to databases and data policies: https://fairsharing.org
GeoCASe—: Geosciences Collection Access Service: http://www.geocase.eu
GBOL—: German Barcode of Life: https://www.bolgermany.de/
GFBio—: German Federation for Biological Data: https://www.gfbio.org
GFBio—: Concepts and Standards in the Public Wiki of the German Federation for Biological Data: https://gfbio.biowikifarm.net/wiki/Concepts_and_Standards
GGBN—: Global Genome Biodiversity Network: http://www.ggbn.org/ggbn_portal/
GGBN—: LOD compliant Wiki representation: https://terms.tdwg.org/wiki/GGBN_Data_Standard
GGBN Data Standard v1: http://wiki.ggbn.org/ggbn/GGBN_Data_Standard_v1
GSC—: Genomic Standards Consortium with Biodiversity Genomics Working Group: http://gensc.org/projects/biodiversity-genomics-working-group/
iDigBio—: Integrated Digitized Biocollections: https://www.idigbio.org/
INSDC—: International Nucleotide Sequence Database Collaboration: http://www.insdc.org/
LIMS—: Laboratory information management system: https://en.wikipedia.org/wiki/Laboratory_information_management_system
MIxS—: LOD compliant Wiki representation: https://terms.tdwg.org/wiki/MIxS
MIxS—: Minimum Information about any (x) Sequence (MIxS): http://gensc.org/mixs/
MIxS Compliance and implementation: http://gensc.org/mixs/mixs-compliance-and-implementation/
Obolibrary: http://purl.obolibrary.org
SDD—: Structured Descriptive Data: TDWG current (2005) standard: https://www.tdwg.org/standards/sdd/
SKOS—: Simple Knowledge Organization System, Introduction to SKOS: http://www.w3.org/2004/02/skos/intro
SPREC—: Standard preanalytical code V.20 developed by the ISBER Biospecimen Science Working Group: http://www.isber.org/?page=SPREC
TDWG group on Biodiversity Information Standards: http://www.tdwg.org/standards
Wikipedia—: The Free Encyclopedia: https://en.wikipedia.org
